# Distinct Gut Microbiota Profiles Associated with Advanced Hepatocellular Carcinoma in a Thai Cohort: A 16S rRNA Sequencing Study

**DOI:** 10.3390/cancers17172915

**Published:** 2025-09-05

**Authors:** Thanakorn Charoenthanadhol, Jutarop Phetcharaburanin, Theerayut Bubpamala, Aumkhae Sookprasert, Jarin Chindaprasirt, Thanachai Sanlung, Piyakarn Watcharenwong, Siraphong Putraveephong, Kosin Wirasorn

**Affiliations:** 1 Medical Oncology Unit, Department of Medicine, Faculty of Medicine, Khon Kaen University, Khon Kaen 40000, Thailand; modthanakorn@hotmail.com (T.C.);; 2 Department of Systems Biosciences and Computational Medicine, Faculty of Medicine, Khon Kaen University, Khon Kaen 40000, Thailand; 3 Khon Kaen University National Phenome Institute, Office of the President, Khon Kaen University, Khon Kaen 40000, Thailand; 4 Thailand Metabolomics Association, Bangkok 10700, Thailand

**Keywords:** hepatocellular carcinoma, gut microbiota, dysbiosis, 16S rRNA sequencing

## Abstract

**Background**: Hepatocellular carcinoma (HCC) remains a major contributor to global cancer mortality. Increasing evidence suggests that the gut microbiota is a key modulator of liver disease progression and a promising source of non-invasive biomarkers. However, regional disparities in microbial profiles, influenced by ethnicity, diet, and environment, limit the generalizability of the existing data. This study aimed to characterize gut microbiota alterations in Thai patients with advanced untreated HCC compared to healthy individuals. **Method**: Fecal samples from 27 untreated Thai advanced HCC patients were collected, and data from 31 healthy individuals retrieved from a previous study were employed. Gut microbiota profiles were analyzed using 16S rRNA gene sequencing. **Results**: Alpha diversity was significantly reduced in HCC patients (*p* < 0.001). At the phylum level, Proteobacteria and Firmicutes were enriched in HCC, whereas Actinobacteria were more abundant in controls. Proteobacteria and Bacteroidota levels were positively correlated with serum alpha-fetoprotein (AFP) levels, whereas Firmicutes were negatively correlated with ALBI scores. **Conclusions**: Thai patients with advanced HCC exhibited distinct gut microbial signatures, characterized by dysbiosis and expansion of Proteobacteria. These findings support the role of the gut microbiota as a potential non-invasive biomarker for disease severity and prognosis in HCC, underscoring the importance of population-specific microbial studies.

## Simple Summary

Hepatocellular carcinoma (HCC) is one of the most common and deadly cancers worldwide, and new approaches are needed to improve diagnosis and treatment. One promising area of research is the gut microbiota, the community of bacteria that live in the intestines and influence overall health. In this study, we compared Thai patients with advanced HCC to healthy individuals. We found that patients with HCC had lower diversity of gut bacteria, showing an imbalance called dysbiosis. Certain bacterial groups, particularly Proteobacteria, were much more common in HCC patients, while others were reduced. We also observed that some bacterial patterns were linked with blood markers and measures of liver function that reflect disease severity. These results suggest that gut bacteria may play a role in HCC progression and could provide useful, non-invasive indicators of disease status. Because the study included a relatively small number of patients and did not include a separate validation group, the findings should be considered hypothesis-generating. Larger and more comprehensive studies will be needed to confirm these results and explore whether targeting the gut microbiota could improve the care of patients with HCC.

## 1. Introduction

Liver cancer is the sixth most commonly diagnosed cancer and the third leading cause of cancer-related deaths worldwide, with over 900,000 new cases and 830,000 deaths annually estimated [1]. Hepatocellular carcinoma (HCC) is the most common primary liver cancer, accounting for approximately 90% of global HCC cases [2] and 44% of cases in Thailand [3]. Common risk factors for HCC include viral hepatitis, alcohol consumption, and metabolic-associated fatty liver disease (MAFLD). In Asia, including Thailand, HBV infection is the predominant risk factor for HCC, whereas HCV infection is more prevalent in North America and Europe [2,3].

The pathophysiology of HCC is complex and not fully understood. Studies suggest that HCC development results from interactions between multiple factors, including genetic predisposition, viral and non-viral risk factors, the cellular microenvironment, and immune cell activity. Recent preclinical and clinical studies have examined the microbiome–gut–liver axis to better understand the role of gut microbiota in HCC development. Disruptions in the gut microbiota composition can lead to bacterial overgrowth, increased intestinal permeability, bacterial translocation, and endotoxemia, all of which contribute to liver disease progression. Several factors, including diet, alcohol consumption, obesity, infection, inflammation, and genetic predisposition, also play a role in shaping the gut microbiota composition and bile acid metabolism. Dysbiosis, an imbalance in the gut microbiota, fosters a pro-inflammatory environment that stimulates the secretion of cytokines, which are key drivers of HCC initiation and progression [4].

A lack of surveillance often results in late-stage diagnosis, leading to poor prognosis. According to the Barcelona Clinic Liver Cancer (BCLC) 2022 guidelines [5], HCC treatment is stage-dependent. Systemic therapy is the preferred treatment approach in BCLC stage C (advanced stage). First-line options currently include either a combination of immune checkpoint inhibitors with anti-angiogenesis therapy or dual immunotherapy, both of which have demonstrated improvements in median overall survival, although response rates remain relatively low (20–30%) [6,7]. Currently, no predictive biomarkers have been established for HCC. However, several potential biomarkers are under investigation, including the gut microbiome and the tumor microenvironment. A study from Asian countries [8,9,10] analyzed the stool microbiome of patients with HCC and found an increased prevalence of pathogenic bacteria, including members of the phyla Proteobacteria, Firmicutes, Patescibacteria, Bacteroidetes, Actinobacteria, and Fusobacteria. Emerging studies from China suggest that gut microbiota alterations may serve as potential biomarkers for the early diagnosis, prognosis, and prediction of responses to immune checkpoint inhibitors in HCC patients [11,12,13]. Recent research also indicates that fecal microbiota transplantation may help overcome resistance to immune checkpoint inhibitors in solid cancers, including HCC [14,15].

However, there are limited data on the characterization of the gut microbiota in Thai patients with HCC, which may differ from populations in China and other regions. Geography, urbanization, ethnicity, and dietary habits are key factors that influence the composition of the gut microbiota [16]. Region-specific research is essential for capturing relevant microbial signatures and improving local clinical applicability. Our aim was to study gut microbiota alterations at both the phylum and genus levels in patients with HCC and a healthy population in Thailand.

## 2. Methods

### 2.1. Study Design and Subjects

This single-center analytical study was conducted at the Srinagarind Hospital, Khon Kaen University, Thailand. Thirty patients with advanced HCC were recruited from a medical oncology outpatient clinic. Eligible patients had previously untreated advanced HCC (BCLC-C or BCLC-B, who were either unsuitable for or refractory to locoregional treatment) and were scheduled to receive systemic therapy. Additionally, all the included patients were born in the northeastern region of Thailand. The main exclusion criteria were as follows: (1) use of medications that affect gut microbiota composition, including antibiotics, proton pump inhibitors, probiotics, and ursodeoxycholic acid; (2) adherence to a specific dietary pattern; and (3) inability to consume adequate calories from food without medical nutritional support.

The diagnosis of HCC is based on characteristic imaging findings, including arterial phase hyperenhancement with portal venous or delayed phase washout, as observed on contrast-enhanced multiphasic CT or MRI in patients with cirrhosis or through histological confirmation [17]. HBV-related HCC was defined by serum HBsAg positivity, whereas HCV-related HCC was defined by serum anti-HCV positivity.

Fecal specimens from HCC patients were collected and analyzed in this study, while control group data were obtained from the previously published “CASCAP19: HE571283” project conducted by Khon Kaen University [18]. The healthy control group consisted of individuals without underlying diseases who had normal bile duct findings and normal liver parenchyma based on ultrasonographic screening under the Cholangiocarcinoma Screening and Care Program (CASCAP) [19] at Srinagarind Hospital, Faculty of Medicine, Khon Kaen University, Thailand.

Subsequently, we analyzed the correlation between gut microbiota composition and key clinical prognostic parameters in patients with HCC, including tumor size, alpha-fetoprotein (AFP) levels, portal vein invasion, extrahepatic metastasis, Child–Pugh score, ALBI score, and 12-month survival status.

### 2.2. Fecal DNA Extraction and 16S rRNA Sequencing

Genomic DNA was extracted from 200 mg of fecal samples using the ZymoBIOMICS™ DNA Miniprep Kit (Zymo Research, Tustin, CA, USA), following the manufacturer’s protocol optimized for microbial DNA recovery. Samples were homogenized using ZR BashingBead™ Lysis Tubes, and DNA was purified through column-based steps and further cleaned using a Zymo-Spin™ III-HRC filter to remove PCR inhibitors. The DNA concentration was measured, and samples were stored at −20 °C until sequencing.

Full-length 16S rRNA gene amplification and library preparation were performed using the Oxford Nanopore Technologies (ONT) 16S Barcoding Kit (SQK-16S024) (Oxford Nanopore Technologies plc, Oxford, UK), following the standard protocol. Barcoded libraries were pooled and sequenced on a MinION device using an R9.4.1 flow cell (FLO-MIN106). Raw ONT reads were based on Dorado basecaller with the super-accuracy model dna_r9.4.1_e8_sup@v3.6. During this step, adapter trimming, barcode trimming, and quality filtering were applied to retain only the reads with a Q score above 7.

Illumina (Illumina, Inc., San Diego, CA, USA) paired-end reads were processed using the DADA2 pipeline (version 1.26.0) in the R environment. This process included quality filtering and trimming of low-quality bases and adapters, dereplication of identical reads, error-rate learning, sample inference using the DADA2 algorithm, merging of paired-end reads, and removal of chimeric sequences.

### 2.3. Bioinformatic Analysis and Taxonomic Classification

Following quality control, high-quality reads from both platforms were aligned and taxonomically classified using Minimap2 (https://github.com/lh3/minimap2, accessed on 6 August 2024) with parameters optimized for long- or short-read data. ONT reads were processed using Minimap2 in long-read mode (-x map-ont), whereas Illumina reads were processed separately using short-read mode (-x sr) to account for platform-specific alignment characteristics. Taxonomic assignment was performed using the SILVA 138.1 reference database. To correct for batch effects and differences in sequencing depth between platforms, taxonomic profiles were normalized by calculating the relative abundance (in percentages) of each taxon per sample. All sequencing procedures and bioinformatic analyses, including platform-specific preprocessing, alignment, taxonomic classification, and normalization, were conducted by the Khon Kaen University National Phenome Institute.

### 2.4. Statistical Analysis

A priori sample size estimation indicated that a minimum of 55 participants across both groups would provide 80% power to detect statistically significant differences, with a two-sided alpha level of 0.05. This estimate was based on the expected effect sizes reported in previous microbiota studies involving hepatocellular carcinoma and gut microbial diversity.

The microbiota profile, including biodiversity indices and relative abundances, was summarized for each group. Continuous variables are presented as mean ± standard deviation (SD) if normally distributed or as median and interquartile range (IQR) if non-normally distributed. Normality was assessed using the Shapiro–Wilk test. For group comparisons, Student’s *t*-test was used for normally distributed variables and the Wilcoxon rank-sum test for non-normally distributed variables. Categorical variables were analyzed using the chi-squared test or Fisher’s exact test, as appropriate. Correlations between clinical prognostic parameters (e.g., etiology, tumor size, AFP level, portal vein invasion, and Child–Pugh class) and the relative abundances of the top 10 phyla and top 20 genera in the HCC group were assessed using Spearman’s rank correlation analysis.

All statistical analyses were conducted using RStudio (version 2024.12.1) and R software (version 4.4.3). The MicrobiomeAnalyst platform (www.microbiomeanalyst.ca) was used for data preprocessing, normalization, alpha and beta diversity analysis, and taxonomic visualization. All statistical tests were two-sided, and a *p*-value < 0.05 was considered statistically significant.

## 3. Results

### 3.1. Demographic Data

Thirty eligible patients with advanced HCC were recruited, and fecal specimens were collected for microbial analysis. Three samples were excluded due to unsuccessful genomic profiling following DNA extraction, resulting in 27 patients being included in the final analysis. Microbial data from 31 healthy individuals were analyzed as a control group.

The demographic and clinical characteristics of the patients with HCC are summarized in Table 1. The majority of the patients were male (77.8%), with a median age of 64 years. Among the participants, 66.7% (15 patients) were classified as BCLC stage C and 44.4% presented with extrahepatic metastasis. Prior locoregional treatment had been administered to 40% of the patients. The median alpha-fetoprotein (AFP) level was 307 ng/mL (range, 15.10 to 23,240.00 ng/mL).

### 3.2. Alpha and Beta Diversity Analyses

Analysis of alpha diversity indices revealed a significant reduction in microbial diversity in patients with advanced HCC compared to that in the control group (*p* < 0.001 for all indices). Specifically, richness was lower in patients with HCC than in controls (182.92 vs. 276.48). Similarly, Shannon diversity index was lower in the HCC group (3.19 vs. 3.70). In addition, the Simpson index was higher in patients with HCC (0.085 vs. 0.044), consistent with reduced microbial diversity. The results are summarized in Table 2. Statistical significance was determined using the Wilcoxon rank-sum test.

**Figure 1:**
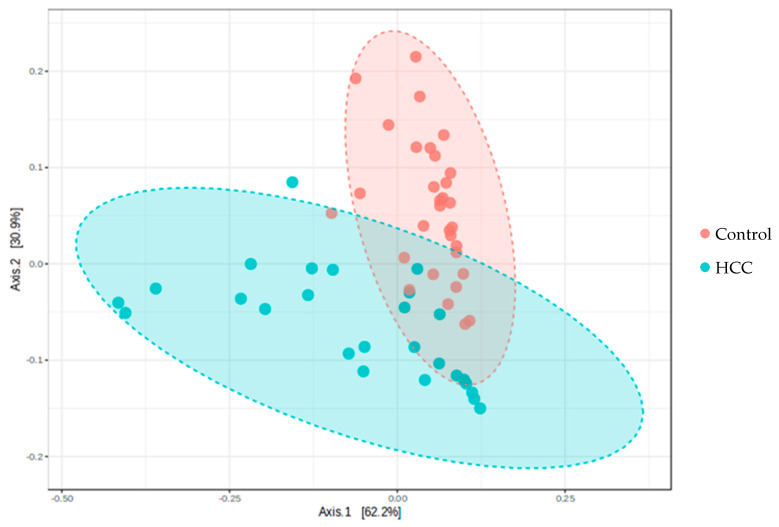
Principal Coordinate Analysis (PCoA) reveals distinct microbial community structures in advanced hepatocellular carcinoma (HCC) patients and healthy controls. The analysis demonstrates clear clustering of the two groups, indicating a significant difference in microbial composition (PERMANOVA, *p* < 0.01).

Beta diversity analysis, performed using Principal Coordinate Analysis (PCoA), revealed a distinct separation between the HCC and control groups (PERMANOVA *p* < 0.01; Figure 1). The first principal coordinate (PCo1) accounted for 62.2% of the variance, while the second principal coordinate (PCo2) accounted for 30.9%, indicating that the majority of the variation in microbial composition was well represented in the plot. This finding suggests that patients with advanced HCC harbor a significantly different gut microbial community from healthy individuals.

### 3.3. Gut Microbiota Shifts in Advanced HCC

**Figure 2:**
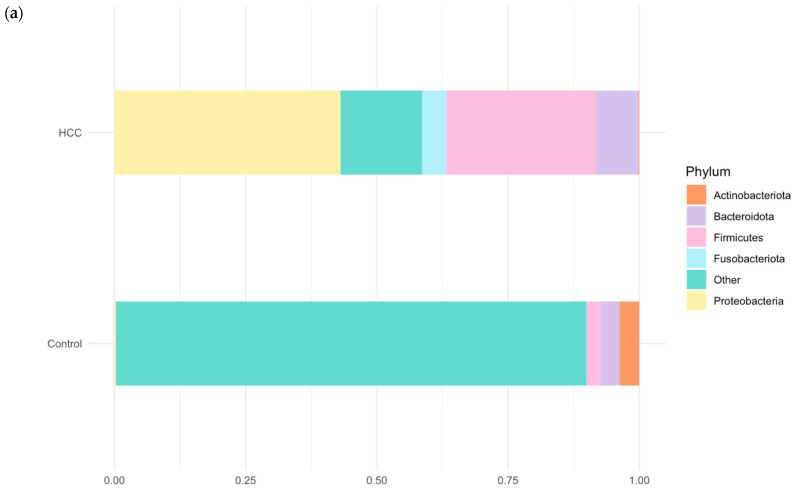

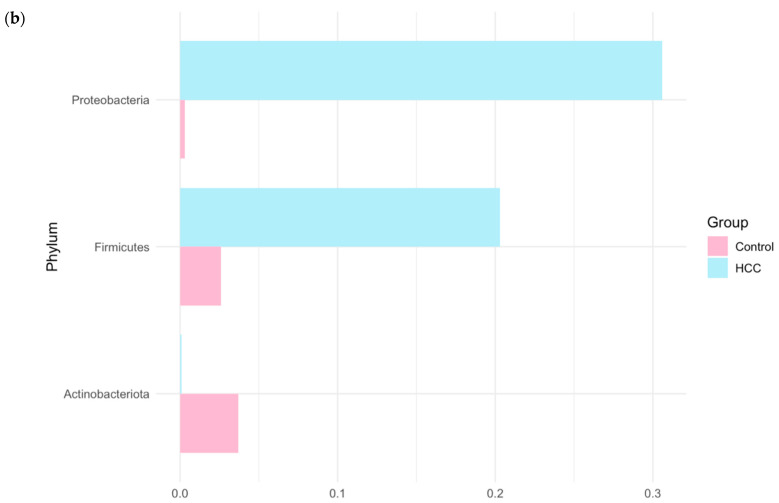
(**a**) Relative abundance of gut microbiota at the phylum level in patients with advanced HCC (*n* = 27) and healthy controls (*n* = 31). Bars represent the median relative abundance of each phylum. (**b**) Statistically significant differences in the relative abundance of major phyla between HCC and control groups (*p* < 0.001), assessed using the Wilcoxon rank-sum test.

At the phylum level, the gut microbiota of patients with advanced HCC was predominantly composed of Proteobacteria (30.6%), Firmicutes (20.3%), Bacteroidota (5.7%), and Fusobacteria (3.3%). In contrast, the healthy control group exhibited higher relative abundances of Actinobacteria (3.7%), Bacteroidota (3.5%), and Patescibacteria (3.2%). The median relative abundance of each phylum is shown in Figure 2a. Comparative analysis revealed that Proteobacteria and Firmicutes were significantly enriched in the HCC group relative to the controls (*p* < 0.001), whereas Actinobacteria were significantly more abundant in the control group (*p* < 0.001). The phylum-level differences in the microbial composition are shown in Figure 2b.

At the genus level, the most prevalent bacterial taxa in the HCC group were Stenotrophomonas (0.5%), Phascolarctobacterium (0.5%), Blautia (0.4%), Ruminococcus (0.4%), and Butyricicoccus (0.4%). Conversely, the control group exhibited higher relative abundance of Butyrivibrio (0.2%), Collinsella (0.2%), Holdemanella (0.2%), Enterorhabdus (0.2%), and unclassified members of Lachnospiraceae (0.2%). Statistical comparisons revealed that several genera were significantly enriched in the HCC group (*p* < 0.001), including Stenotrophomonas, Phascolarctobacterium, Blautia, Ruminococcus, Butyricicoccus, Lachnoclostridium, Flavonifractor, Streptococcus, and Lachnospira. These genera have been implicated in the microbial dysbiosis and inflammatory responses associated with liver diseases. Detailed comparisons of relative abundances at both the phylum and genus levels are summarized in Supplementary Tables S1 and S2, respectively.

### 3.4. Correlation Between Gut Microbiota and Clinical Prognostic Factors

**Figure 3:**
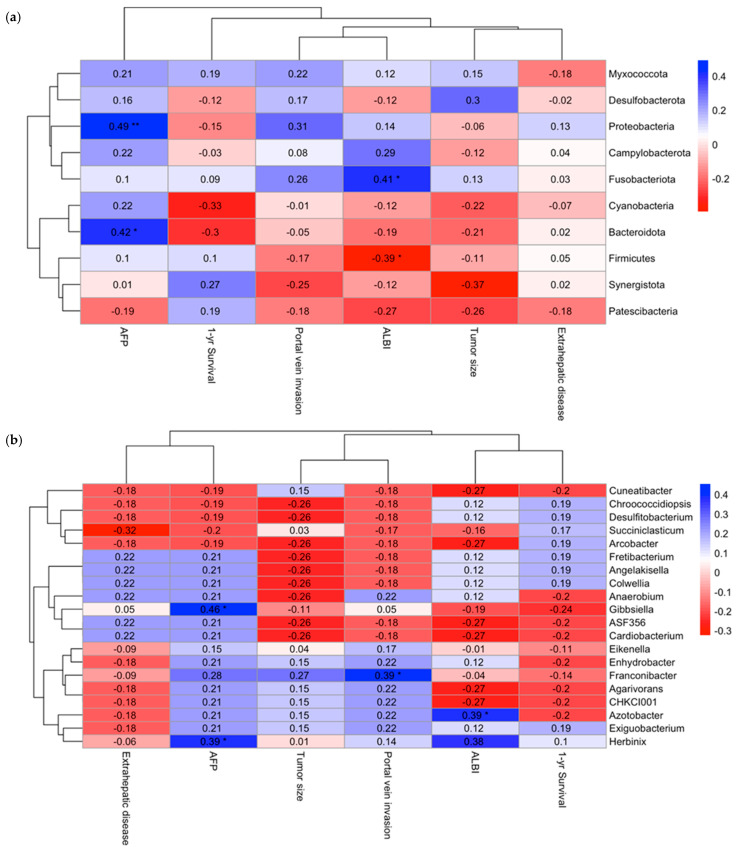
(**a**) Heatmap illustrating Spearman’s correlation between gut microbiota at the phylum level and clinical prognostic factors in patients with advanced hepatocellular carcinoma (HCC). (**b**) Heatmap illustrating Spearman’s correlation between gut microbiota at the genus level and clinical prognostic factors in patients with advanced HCC. The Spearman correlation coefficients (ρ) are shown within each cell of the heatmap. * indicates *p* < 0.05; ** indicates *p* < 0.01. The color intensity reflects the strength and direction of the correlation, with blue representing positive correlations and red representing negative correlations.

Spearman correlation analysis was conducted to explore the associations between gut microbial composition and key clinical prognostic parameters in patients with advanced HCC, including tumor size, serum alpha-fetoprotein (AFP) levels, portal vein invasion, extrahepatic metastasis, ALBI score, and 12-month survival status. At the phylum level, Proteobacteria (ρ = 0.49, *p* < 0.01) and Bacteroidota (ρ = 0.42, *p* < 0.05) demonstrated significant positive correlations with elevated AFP levels, while Firmicutes (ρ = −0.39, *p* < 0.05) were negatively correlated with the ALBI score, indicating a potential link between microbial composition and liver function or disease severity (Figure 3a).

At the genus level, Gibbsiella (ρ = 0.46, *p* < 0.05) and Herbinix (ρ = 0.39, *p* < 0.05) were positively associated with elevated AFP levels. Additionally, Franconibacter (ρ = 0.39, *p* < 0.05) showed a significant positive correlation with the presence of portal vein invasion, whereas Azotobacter (ρ = −0.39, *p* < 0.05) was inversely correlated with the ALBI score (Figure 3b). These findings highlight the potential relationship between specific gut microbial taxa and clinical markers of tumor burden and liver function in advanced HCC. Detailed association between microbial taxa and clinical prognostic factors including AFP and ALBI scores are presented in Supplementary Tables S3 and S4.

## 4. Discussion

Our study demonstrated significant alterations in the gut microbiota composition of patients with hepatocellular carcinoma (HCC). Microbial diversity, as assessed by alpha diversity indices, was significantly reduced in the HCC group compared to that in healthy controls, which is consistent with previous studies indicating gut microbiota dysbiosis in HCC [9,20]. At the phylum level, Proteobacteria and Firmicutes were significantly more abundant in patients with HCC, while Actinobacteria were enriched in the control group. The high abundance of Proteobacteria in patients with HCC observed in this study aligns with reports from Europe [20], China [21,22], Korea [23], and Thailand [10]. The relative abundance of Proteobacteria in the HCC group (~30.6%) was markedly higher than the ~1–5% typically reported in healthy gut microbiota [24,25], reinforcing its potential role as a microbial marker of gut–liver axis disruption in hepatocarcinogenesis. Proteobacteria are known to produce lipopolysaccharides (LPS), pro-inflammatory molecules that can translocate across a compromised intestinal barrier, activate the gut–liver axis, and trigger systemic inflammation and hepatic immune responses, processes implicated in liver injury and hepatocarcinogenesis [26]. Expansion of Proteobacteria has been recognized as a signature of microbial dysbiosis. Emerging evidence also suggests that Proteobacteria-enriched microbiota may influence the treatment response, including potential resistance to immunotherapy in HCC [27]. Consequently, modulation of the gut microbiome has emerged as a potential adjunct to current HCC treatments [28,29]. However, further studies are needed to establish its prognostic and therapeutic relevance, ideally through multicenter collaborations in diverse geographic regions and with consideration of lifestyle factors, including alcohol consumption. Findings related to a higher abundance of Firmicutes and a lower abundance of Actinobacteria in HCC remain inconsistent across published studies [8,20,21,23]. These results should be interpreted as hypothesis-generating, providing a foundation for further investigation rather than a definitive conclusion.

The enrichment of genera such as Streptococcus and Lachnoclostridium in patients with HCC observed in our study is consistent with previous reports, supporting the concept that gut dysbiosis contributes to liver disease progression [20,30]. These genera are frequently linked to pro-inflammatory states and have been associated with advanced liver disease and carcinogenesis. Conversely, we also observed an increased abundance of butyrate-producing bacteria, such as *Butyricicoccus*, which may reflect a compensatory response to microbial imbalance, a finding that has been inconsistently reported across studies. Variations in gut microbiota composition across studies at both the phylum and genus levels may be attributable to differences in host population characteristics, regional dietary habits, underlying disease etiology, sample sizes, and sequencing platforms or analytical methods.

Beyond taxonomic profiling, our findings suggest that specific gut microbial patterns are associated with clinical indicators of disease severity in patients with advanced HCC. Proteobacteria and Bacteroidota were positively correlated with AFP levels, whereas Firmicutes were negatively associated with ALBI score. These correlations may reflect gut–liver axis interactions. Enrichment of Proteobacteria and Bacteroidota, both linked to inflammation and microbial translocation, may contribute to elevated AFP levels. In contrast, reduced Firmicutes, rich in SCFA-producing, anti-inflammatory taxa, may be associated with poorer liver function, as indicated by higher ALBI scores. These findings are consistent with those of prior studies implicating the gut microbiota in hepatic immune regulation and tumor progression [31]. Importantly, the observed microbial associations with AFP and ALBI highlight their potential utility as clinically relevant biomarkers. Beyond prognostic value, these microbial signatures may also represent novel therapeutic targets, with microbiome modulation offering a promising adjunctive strategy to enhance immunotherapy efficacy and overcome resistance to immune checkpoint inhibitors in advanced HCC [32,33,34].

This study is one of the first comprehensive reports on gut microbiota alterations in Thai patients with advanced HCC. We specifically investigated a cohort of treatment-naïve, advanced-stage HCC patients, minimizing confounding factors, such as prior therapy or microbiota-altering medications. Moreover, our study provides clinically relevant insights by integrating microbial diversity measures, taxonomic profiling, and correlations with key prognostic factors, such as AFP level and ALBI score. These strengths establish this dataset as a robust reference for gut microbiota patterns of advanced HCC in the Thai population.

Our study has a few limitations. First, the relatively small sample size may limit the generalizability of our findings, although similar sample sizes have yielded meaningful results in prior HCC microbiome studies [8,9]. Second, the absence of a validation cohort further restricts external validity, highlighting the need for future multicenter studies that incorporate independent validation sets. Third, differences in sequencing platforms were present between groups; however, benchmarking studies [35,36] have demonstrated that ONT and Illumina platforms produce broadly comparable results at higher taxonomic levels, despite some discrepancies at finer resolutions. While standardized workflows and data normalization were applied to reduce such variability, platform-specific biases cannot be completely excluded.

Our study underscores the importance of building local microbiome datasets. This investigation represents a key first step in characterizing the gut microbiota of patients with advanced-stage HCC in Thailand. Larger multicenter longitudinal studies with standardized sequencing platforms, serial fecal sampling, and clinical follow-up will be essential to validate these microbial signatures, elucidating their mechanistic links to the gut–liver axis, and determine their prognostic or therapeutic relevance in HCC.

## 5. Conclusions

This study provides novel insights into the gut microbiota landscape of Thai patients with advanced HCC, revealing significant dysbiosis characterized by reduced microbial diversity and distinct taxonomic alterations. Notably, the overrepresentation of Proteobacteria and depletion of Actinobacteria distinguished patients with HCC from healthy controls, highlighting the potential microbial signatures associated with hepatic carcinogenesis. Furthermore, correlations between specific microbial taxa and clinical prognostic markers, such as AFP levels and ALBI scores, underscore the potential of the gut microbiota as non-invasive indicators for disease severity and outcome prediction.

These findings emphasize the importance of region-specific microbiome profiling in understanding host–microbial interactions within the context of liver cancer. The integration of microbial signatures with clinical phenotypes may pave the way for microbiota-informed precision medicine strategies for HCC management. Future prospective studies with larger cohorts and multi-omics integration are warranted to validate these associations and explore the therapeutic implications of modulating the gut microbiota in hepatocellular carcinoma.

## Figures and Tables

**Table 1:** Demographic and clinical characteristics of patients with advanced hepatocellular carcinoma (HCC) included in the study (*n* = 27).

Characteristics	HCC Group (N = 27)
Gender	
Male	21 (77.8%)
Female	6 (22.2%)
Age, median (range)	64 (48–78)
BMI (kg/m^2^)	21.84 ± 3.61
ECOG performance scale, *n* (%)	
0	15 (55.6%)
1	11 (40.7%)
2	1 (3.7%)
BCLC staging, *n* (%)	
B	9 (33.3%)
C	18 (66.7%)
Viral hepatitis, *n* (%)	
B	8 (29.6%)
C	14 (51.9%)
Non-viral	5 (18.5%)
Previous local treatment, *n* (%)	12 (40%)
Child–Pugh score, *n* (%)	
A (5)	15 (55.6%)
A (6)	10 (37.0%)
B (7)	1 (3.7%)
B (8)	1 (3.7%)
ALBI score, *n* (%)	
1	9 (33.3%)
2	17 (63.0%)
3	1 (3.7%)
Median AFP level (IQR)	307.000 (15.10–23,240.00)
AFP ≥ 400, *n* (%)	13 (48.1%)
AFP < 400, *n* (%)	14 (51.9%)
Mean tumor size (±SD)	7.73 ± 4.27
Tumor size > 5 cm, *n* (%)	17 (63.0%)
Tumor size ≤ 5 cm, *n* (%)	10 (37.0%)
Extrahepatic metastasis, *n* (%)	12 (44.4%)

Abbreviation: BMI, Body mass index; ECOG, the Eastern Cooperative Oncology Group; BCLC, Barcelona Clinic Liver Cancer; ALBI, Albumin-bilirubin; AFP, Alpha-fetoprotein.

**Table 2:** Comparison of microbial alpha diversity indices in advanced hepatocellular carcinoma (HCC) patients (*n* = 27) and healthy controls (*n* = 31). Richness, Shannon diversity index, and Simpson’s index were used to assess within-sample microbial diversity. Statistical significance was determined using the Wilcoxon rank-sum test.

Diversity Index	HCC Group	Control Group	*p*-Value
Richness	182.92	276.48	<0.001
Shannon diversity index	3.19	3.70	<0.001
Simpson’s index	0.085	0.044	<0.001

## Data Availability

Owing to ethical and privacy restrictions, the raw data are not publicly available but may be obtained from the corresponding author upon reasonable request and with appropriate institutional approval.

## Supplementary Materials

The following supporting information can be downloaded at: https://www.mdpi.com/article/10.3390/cancers17172915/s1, Table S1: Comparison of the relative abundance of gut microbiota between hepatocellular carcinoma (HCC) patients (*n* = 27) and healthy controls (*n* = 31) at the phylum level; Table S2: Comparison of the relative abundance of gut microbiota between hepatocellular carcinoma (HCC) patients (*n* = 27) and healthy controls (*n* = 31) at the genus level; Table S3: Spearman’s correlation between gut microbiota at the phylum level and clinical prognostic factors in patients with advanced hepatocellular carcinoma (HCC); Table S4: Spearman’s correlation between gut microbiota at the genus level and clinical prognostic factors in patients with advanced hepatocellular carcinoma (HCC).
