# Supplementary material for: Distinct Gut Microbiota Profiles Associated with Advanced Hepatocellular Carcinoma in a Thai Cohort: A 16S rRNA Sequencing Study
Source: Cancers (Basel). 2025 Sep 5;17(17):2915. doi: 10.3390/cancers17172915 (PMC12428426; doi:10.3390/cancers17172915)
Supplement: Supplementary file 1 [file cancers-17-02915-s001.zip › cancers-3784951-supplementary.pdf]

Supplementary Data

Table S1. Comparison of the relative abundance of gut microbiota between hepatocellular carcinoma (HCC) patients (*n* = 27) and healthy controls (*n* = 31) at the phylum level. Values represent the median relative abundance of each phylum in both groups. Statistical significance was assessed using the Wilcoxon rank-sum test.

Phylum	HCC group	Control group	p-value
Proteobacteria	0.306(0.008-0.041)	0.004(0.001-0.007)	<.001
Firmicutes	0.203(0.012-0.021)	0.026(0.014-0.019)	<.001
Bacteroidota	0.057(0.003-0.006)	0.035(0.016-0.033)	.107
Fusobacteriota	0.033(0.00-0.017)	0.001(0.00-0.003)	.367
Actinobacteriota	0.001(0.00-0.00)	0.037(0.013-0.045)	<.001

Table S2. Comparison of the relative abundance of gut microbiota between hepatocellular carcinoma (HCC) patients (*n* = 27) and healthy controls (*n* = 31) at the genus level. Values represent the median relative abundance of each genus in both groups. Statistical significance was assessed using the Wilcoxon rank-sum test.

Genus	HCC group	Control group	p-value
Stenotrophomonas	0.006(0.0026-0.014)	0.00(0.00-0.00)	<.001
Granulicatella	0.004(0.00-0.008)	0.00(0.000-0.001)	.035
Faecalibacterium	0.003(0.00-0.009)	0.001(0.000-0.004)	.121
Fusicatenibacter	0.004(0.00-0.011)	0.001(0.000-0.002)	.094
Ruminococcus	0.004(0.001-0.007)	0.001(0010-0.002)	<.001
Blautia	0.004(0.002-0.007)	0.002(0.00-0.003)	.002
Phascolarctobacterium	0.005(0.001-0.012)	0.001(0.00-0.002)	.010
Butyricicoccus	0.004(0.002-0.008)	0.002(0.001-0.003)	.005
Dorea	0.003(0.00-0.008)	0.002(0.001-0.003)	.239
Colidextribacter	0.002(0.00-0.006)	0.001(0.00-0.003)	.538
Eubacterium	0.003(0.001-0.006)	0.002(0.001-0.003)	.103
Streptococcus	0.002(0.001-0.010)	0.000(0.00-0.001)	<.001
Roseburia	0.002(0.001-0.006)	0.001(0.00-0.002)	.110
Escherichia-Shigella	0.001(0.00-0.008)	0.00(0.00-0.00)	<.001
Flavonifractor	0.003(0.001-0.009)	0.001(0.00-0.001)	.009
Lachnoclostridium	0.003(0.002-0.006)	0.001(0.001-0.002)	<.001
Enterobacter	0.002(0.00-0.006)	0.00(0.00-0.001)	<.001
Haemophilus	0.002(0.00-0.004)	0.00(0.00-0.00)	.001

Lachnospira	0.002(0.00-0.009)	0.00(0.00-0.001)	.018

Table S3. Spearman's correlation between gut microbiota at the phylum level and clinical prognostic factors in patients with advanced hepatocellular carcinoma (HCC).
Each cell displays the Spearman correlation coefficient (ρ), with the corresponding *p*-value shown in parentheses below the coefficient.

Phylum	Five	PortalVein	Extrahepatic	AFP	ALBI	1-yr survival status
Proteobacteria<br>(p-value)	-0.0591<br>(0.7697)	0.3062<br>(0.1203)	0.1340<br>(0.5053)	0.4938<br>(0.0089)	0.1409<br>(0.4834)	-0.1523<br>(0.4483)
Campylobacterota<br>(p-value)	-0.1219<br>(0.5448)	0.0790<br>(0.6954)	0.0395<br>(0.8450)	0.2191<br>(0.2723)	0.2921<br>(0.1393)	-0.0262<br>(0.8969)
Fusobacteriota<br>(p-value)	0.1325<br>(0.5099)	0.2576<br>(0.1945)	0.0297<br>(0.8830)	0.0977<br>(0.6277)	0.4136<br>(0.0320)	0.0887<br>(0.6600)
Firmicutes<br>(p-value)	-0.1083<br>(0.5907)	-0.1723<br>(0.3903)	0.0478<br>(0.8127)	0.1010<br>(0.6163)	-0.3930<br>(0.0426)	0.1047<br>(0.6033)
Myxococcota<br>(p-value)	0.1504<br>(0.4539)	0.2193<br>(0.2718)	-0.1754<br>(0.3815)	0.2147<br>(0.2822)	0.1192<br>(0.5538)	0.1890<br>(0.3451)
Cyanobacteria<br>(p-value)	-0.2227<br>(0.2641)	-0.0053<br>(0.9792)	-0.0686<br>(0.7338)	0.2175<br>(0.2759)	-0.1207<br>(0.5485)	-0.3255<br>(0.0976)
Bacteroidota<br>(p-value)	-0.2068<br>(0.3007)	-0.0478<br>(0.8127)	0.0191<br>(0.9245)	0.4150<br>(0.0313)	-0.1929<br>(0.3351)	-0.2950<br>(0.1352)
Desulfobacterota<br>(p-value)	0.3003<br>(0.1280)	0.1677<br>(0.4032)	-0.0248<br>(0.9021)	0.1592<br>(0.4277)	-0.1209<br>(0.5479)	-0.1235<br>(0.5394)
Synergistota<br>(p-value)	-0.3685<br>(0.0586)	-0.2528<br>(0.2033)	0.0211<br>(0.9169)	0.0099<br>(0.9608)	-0.1177<br>(0.5588)	0.2724<br>(0.1693)
Patescibacteria<br>(p-value)	-0.2557<br>(0.1980)	-0.1754<br>(0.3815)	-0.1754<br>(0.3815)	-0.1861<br>(0.3528)	-0.2681<br>(0.1763)	0.1890<br>(0.3451)

Table S4. Spearman's correlation between gut microbiota at the genus level and clinical prognostic factors in patients with advanced hepatocellular carcinoma (HCC).
Each cell displays the Spearman correlation coefficient (ρ), with the corresponding *p*-value shown in parentheses below the coefficient.

Genus	Five	PortalVein	Extrahepatic	AFP	ALBI	1-yr survival status
Franconibacter<br>(p-value)	0.2707<br>(0.1720)	0.3947<br>(0.0416)	-0.0877<br>(0.6636)	0.2789<br>(0.1589)	-0.0351<br>(0.8621)	-0.1396<br>(0.4875)
Succiniclasticum<br>(p-value)	0.0338<br>(0.8669)	-0.1710<br>(0.3938)	-0.3223<br>(0.1011)	-0.1951<br>(0.3295)	-0.1564<br>(0.4360)	0.1701<br>(0.3964)
ASF356<br>(p-value)	-0.2557<br>(0.1980)	-0.1754<br>(0.3815)	0.2193<br>(0.2718)	0.2147<br>(0.2822)	-0.2681<br>(0.1763)	-0.2035<br>(0.3086)
Agarivorans<br>(p-value)	0.1504<br>(0.4539)	0.2193<br>(0.2718)	-0.1754<br>(0.3815)	0.2147<br>(0.2822)	-0.2681<br>(0.1763)	-0.2035<br>(0.3086)
Anaerobium<br>(p-value)	-0.2557<br>(0.1980)	0.2193<br>(0.2718)	0.2193<br>(0.2718)	0.2147<br>(0.2822)	0.1192<br>(0.5538)	-0.2035<br>(0.3086)
Angelakisella<br>(p-value)	-0.2557<br>(0.1980)	-0.1754<br>(0.3815)	0.2193<br>(0.2718)	0.2147<br>(0.2822)	0.1192<br>(0.5538)	0.1890<br>(0.3451)
Arcobacter<br>(p-value)	-0.2557<br>(0.1980)	-0.1754<br>(0.3815)	-0.1754<br>(0.3815)	-0.1861<br>(0.3528)	-0.2681<br>(0.1763)	0.1890<br>(0.3451)
Azotobacter<br>(p-value)	0.1504<br>(0.4539)	0.2193<br>(0.2718)	-0.1754<br>(0.3815)	0.2147<br>(0.2822)	0.3873<br>(0.0459)	-0.2035<br>(0.3086)
CHKCI001<br>(p-value)	0.1504<br>(0.4539)	0.2193<br>(0.2718)	-0.1754<br>(0.3815)	0.2147<br>(0.2822)	-0.2681<br>(0.1763)	-0.2035<br>(0.3086)
Cardiobacterium<br>(p-value)	-0.2557<br>(0.1980)	-0.1754<br>(0.3815)	0.2193<br>(0.2718)	0.2147<br>(0.2822)	-0.2681<br>(0.1763)	-0.2035<br>(0.3086)
Chroococcidiopsis<br>(p-value)	-0.2557<br>(0.1980)	-0.1754<br>(0.3815)	-0.1754<br>(0.3815)	-0.1861<br>(0.3528)	0.1192<br>(0.5538)	0.1890<br>(0.3451)
Colwellia<br>(p-value)	-0.2557<br>(0.1980)	-0.1754<br>(0.3815)	0.2193<br>(0.2718)	0.2147<br>(0.2822)	0.1192<br>(0.5538)	0.1890<br>(0.3451)
Cuneatibacter<br>(p-value)	0.1504<br>(0.4539)	-0.1754<br>(0.3815)	-0.1754<br>(0.3815)	-0.1861<br>(0.3528)	-0.2681<br>(0.1763)	-0.2035<br>(0.3086)
Desulfitobacterium<br>(p-value)	-0.2557<br>(0.1980)	-0.1754<br>(0.3815)	-0.1754<br>(0.3815)	-0.1861<br>(0.3528)	0.1192<br>(0.5538)	0.1890<br>(0.3451)
Eikenella<br>(p-value)	0.0361<br>(0.8581)	0.1666<br>(0.4061)	-0.0877<br>(0.6636)	0.1549<br>(0.4405)	-0.0093<br>(0.9634)	-0.1134<br>(0.5733)

Genus	Five	PortalVein	Extrahepatic	AFP	ALBI	1-yr survival status
Enhydrobacter (p-value)	0.1504 (0.4539)	0.2193 (0.2718)	-0.1754 (0.3815)	0.2147 (0.2822)	0.1192 (0.5538)	-0.2035 (0.3086)
Exiguobacterium (p-value)	0.1504 (0.4539)	0.2193 (0.2718)	-0.1754 (0.3815)	0.2147 (0.2822)	0.1192 (0.5538)	0.1890 (0.3451)
Fretibacterium (p-value)	-0.2557 (0.1980)	-0.1754 (0.3815)	0.2193 (0.2718)	0.2147 (0.2822)	0.1192 (0.5538)	0.1890 (0.3451)
Gibbsiella (p-value)	-0.1116 (0.5795)	0.0465 (0.8179)	0.0465 (0.8179)	0.4550 (0.0171)	-0.1883 (0.3470)	-0.2388 (0.2303)
Herbinix (p-value)	0.0090 (0.9644)	0.1403 (0.4853)	-0.0614 (0.7611)	0.3862 (0.0466)	0.3752 (0.0538)	0.1046 (0.6036)
